# Tree water status and growth of saplings and mature Norway spruce (*Picea abies*) at a dry distribution limit

**DOI:** 10.3389/fpls.2015.00703

**Published:** 2015-09-07

**Authors:** Walter Oberhuber, Albin Hammerle, Werner Kofler

**Affiliations:** ^1^Institute of Botany, Leopold-Franzens-University of InnsbruckInnsbruck, Austria; ^2^Institute of Ecology, Leopold-Franzens-University of InnsbruckInnsbruck, Austria

**Keywords:** dendrometer, dry inner Alpine valley, maximum daily shrinkage, *Picea abies*, radial growth, stem water deficit, wavelet analysis

## Abstract

We evaluated the size effect on stem water status and growth in Norway spruce (*Picea abies* (L.) Karst.) occurring at the edge of its natural range in a dry inner Alpine environment (750 m asl, Tyrol, Austria). Intra-annual dynamics of stem water deficit (ΔW), maximum daily shrinkage (MDS), and radial growth (RG) were compared among saplings (stem diameter/height: 2.2 cm/93 cm; *n* = 7) and mature adult trees (25 cm/12.7 m; *n* = 6) during 2014. ΔW, MDS, and RG were extracted from stem diameter variations, which were continuously recorded by automatic dendrometers and the influence of environmental drivers was evaluated by applying moving correlation analysis (MCA). Additionally, we used Morlet wavelet analysis to assess the differences in cyclic radial stem variations between saplings and mature trees. Results indicate that saplings and mature trees were experiencing water limitation throughout the growing season. However, saplings exhibited a more strained stem water status and higher sensitivity to environmental conditions than mature trees. Hence, the significantly lower radial increments in saplings (0.16 ± 0.03 mm) compared to mature trees (0.54 ± 0.14 mm) is related to more constrained water status in the former, affecting the rate and duration of RG. The wavelet analysis consistently revealed more distinct diurnal stem variations in saplings compared to mature trees. Intra-annual RG was most closely related to climate variables that influence transpiration, i.e., vapor pressure deficit, relative air humidity, and air temperature. MCA, however, showed pronounced instability of climate–growth relationships, which masked missing temporal or significant correlations when the entire study period (April–October) was considered. We conclude that an increase in evaporative demand will impair regeneration and long-term stability of drought-prone inner Alpine Norway spruce forests.

## Introduction

Norway spruce (*Picea abies* (L.) Karst.) is the most widespread coniferous species in the Central European Alps ranging from low elevation to the timberline (Ellenberg and Leuschner, 2010). This tree is moderately shade-tolerant, dominates in late successional stages and shows high sensitivity to soil water supply (e.g., Lévesque et al., 2013; van der Maaten-Theunissen et al., 2013). Schuster and Oberhuber (2013a) detected increasing drought sensitivity of mature *P. abies* in recent decades, which was related to a decline in soil moisture availability due to increasing stand density and tree size. Ecophysiological and dendroclimatological studies have shown that size-mediated changes in functional processes and climate–growth relationships occur in forest trees (Mencuccini et al., 2005; Martínez-Vilalta et al., 2007; Mérian and Lebourgeois, 2011). Therefore, size-dependent sensitivity to climate is expected to affect forest structure and succession. Furthermore, the anticipated changes in climate, which include significant warming in the following decades, changes in seasonal precipitation patterns and an increase in both frequency and intensity of severe droughts (IPCC, 2013), underline the need to evaluate differences in intra-annual development of the plant water status in saplings compared to mature adult trees.

Although plant water status is determined primarily by the physical conditions of the air and soil, trees are capable of counterbalancing temporal water deficits by withdrawing water from water storage locations within the tree, namely, the sapwood, the cell walls, and inactive vessels, the mesophyll of needles and the elastic tissues of the bark, i.e., enlarging xylem cells, cambium, phloem, and bark parenchyma (Zimmermann, 1983). It is generally assumed that the reversible diurnal changes in stem size, i.e., stem shrinking and swelling during day and night, respectively, are the result of changing water potential gradients within the plant (e.g., Whitehead and Jarvis, 1981; Herzog et al., 1995; Zweifel et al., 2001; Sevanto et al., 2011). Reversible changes in daily water content of the stem together with irreversible radial stem growth, i.e., cambial cell division and cell enlargement primarily explain stem radius variations during the growing season. The stem radius variation de-trended for growth was determined to be proportional to the water content in the living tissues of the bark and was termed the tree water deficit (ΔW) (Hinckley and Lassoie, 1981; Herzog et al., 1995; Zweifel et al., 2000). According to several authors (Zweifel et al., 2005; Drew et al., 2011; Köcher et al., 2013) ΔW is closely related to drought stress and is mainly determined by a combination of atmospheric and soil conditions (e.g., Zweifel et al., 2005; Ehrenberger et al., 2012). Maximum daily shrinkage (MDS), defined as the difference between the daily maximum and minimum stem diameter, is a measure of the proportion of water taken up at night that is lost from elastic cambial and phloem tissues during the day. Several authors reported that MDS provides information on tree water relations (Zweifel et al., 2000; Intrigliolo and Castel, 2005; Giovannelli et al., 2007). Hence, high resolution dendrometer measurements of diurnal stem diameter variations provide several parameters related to tree water status and enable the determination of radial growth (RG) (e.g., Downes et al., 1999; Deslauriers et al., 2007). ΔW and MDS determined in this study represent indirect measures of plant water status compared to direct methods, such as leaf water potential or relative water content (Jones, 2007). Advantages of the applied indirect method are that (i) dendrometers allow a continuous, non-destructive record of plant water status, and (ii) ΔW and MDS can be determined simultaneously with radial stem growth. Although especially in isohydric plants stomatal control tends to maintain tissue water status stable (homeostatic regulation), indirect measures of water status are regarded to be highly valuable for detecting physiological responses to water deficits (Naor and Cohen, 2003; Zweifel et al., 2005; Jones, 2007). Previous findings revealed that in an old-aged mixed coniferous stand that included *Pinus sylvestris*, *Larix decidua*, and *P. abies*, *P. abies* most strongly exhausted stem water reservoirs during the growing season (Oberhuber et al., 2014). Conversely, shade-tolerance, shallow rooting and isohydric behavior, i.e., early closing of stomata to stabilize water relations (‘drought avoidance’), were suggested to allow *P. abies* to invade *Pinus sylvestris* stands at dry-mesic sites (Anfodillo et al., 1998; Schuster and Oberhuber, 2013b; Leo et al., 2014).

The aims of this study were therefore (i) to compare the seasonal development of tree water status indicators (ΔW, MDS) and growth of co-occurring saplings and mature *P. abies* growing at the distribution limit at a xeric inner Alpine site during the growing season and (ii) to determine environmental factors that are most closely related to the overall variations in parameters of tree water status and radial stem growth. We are not aware of any study comparing dendrometer derived water status indicators throughout the growing season in co-occurring saplings and mature *P. abies* within the same stand, although contrasting development of stem water status is plausible to occur. Specifically in saplings, lower stem water reserves are expected due to thinner bark and smaller sapwood area compared to mature adult trees (Zweifel et al., 2000; Dawes et al., 2014). Conversely, mature dominant trees are exposed to higher solar radiation, vapor pressure deficit (VPD) and wind velocity, which increase transpiration forcing. In accordance with previous findings that smaller trees take advantage of better rooting in the upper moist soil horizon, lower water demand of small trees and more favorable microclimates below canopy (Schuster and Oberhuber, 2013b), we hypothesized that a less distinct stem water deficit (ΔW) is developed throughout the growing season in saplings compared to mature trees. Furthermore, we expected that ΔW, MDS, and growth are most closely related to the climate variables that influence transpiration and that the temporal relationship between environmental variables and stem water indicators and RG exhibit high stability throughout the growing season.

## Materials and Methods

### Study Site

The study site is part of a postglacial rock-slide area situated in the montane belt (c. 750 m asl) within the inner Alpine dry valley of the Inn River (Tyrol, Austria, 47°13′53′′ N, 10°50′51′′ E; Fliri, 1975). The mean annual air temperature is 7.3°C and the mean annual precipitation amounts to 716 mm (1911–2010, Ötz, 812 m asl, 5 km from the study area). The soil is shallow, reaches maximum depth of 0.2 m at the measurement site and consists of unconsolidated, coarse-textured materials with low water holding capacity. It is classified as rendzic leptosol according to the FAO classification system (FAO, 2006). The dominating plant community on xeric sites is an open Spring Heath-Pine wood (Erico-Pinetum sylvestris; Ellenberg and Leuschner, 2010). *P. abies* co-occurs in the canopy under more mesic conditions, i.e., in hollows or on north-facings slopes. At the selected dry-mesic site a mixed stand composed of *Pinus sylvestris*, *P. abies*, and *Larix decidua* is developed, whereby the proportion referred to basal area is 60, 20, and 20%, respectively. Height and canopy coverage of the selected stand amounted to *c*. 13 m and *c*. 70%, respectively (**Table 1**). The study site was slightly facing north and the moderately shade-tolerant *P. abies* rejuvenates naturally under the mixed canopy. The number of saplings <2 m in height amounts to approximately 10 individuals in an area covering 30m × 30m (cf. Schuster and Oberhuber, 2013b). Saplings and mature trees were selected within the same stand covering an area of *c*. 0.5 ha.

**Table 1:** Site description and tree characteristics (A, aspect; CC, canopy coverage; Prz, Protorendzina (rendzic leptosol); Rh, raw humus; Sd, soil depth).

A	Slope (°)	Soil Type	Humus Type	Sd (cm)	CC (%)	Tree height (m)		Stem diameter^1^ (cm)		Bark width (mm)	
						Mature	Saplings	Mature	Saplings	Mature	Saplings
*N*	<5	Prz	Rh	15–20	70	12.7 ± 1.6	0.93 ± 0.5	24.8 ± 4.8	2.2 ± 0.8	3.6 ± 0.5	1.8 ± 0.4

*Mean values ± SD are shown. Tree height, stem diameter and bark width are significantly different between mature trees and saplings at *P* < 0.001 (Students *t*-test).*

*^1^Stem diameter was determined at breast height (1.30 m) and at 15 cm height for mature trees and saplings, respectively.*

### Microclimate Records

During the study period, air temperature, relative air humidity (RH), daily precipitation and solar radiation were collected automatically (ONSET, Pocasset, MA, USA) at 2 m height on an open ridge, i.e., in a non-vegetated area close to the study area (<100 m in linear distance). Additionally, air temperature and RH were recorded below canopy within the selected stand at 2 m height. Measuring intervals for all sensors were set to 30 min and mean daily air temperatures were calculated by averaging all measurements (48 values per day). The VPD in the air was calculated from the hourly means of air temperature and RH using the equation given in Prenger and Ling (2000). Volumetric soil water content (SWC) at the 5–10 cm and 15–20 cm soil depth layers (*n* = 3 sensors per soil depth) was recorded within the selected stand (ThetaProbes Type ML2x, Delta-T, Cambridge, England). Additionally, soil temperature in the top 5–10 cm soil depth layer was measured (S-TMB, ONSET, Pocasset, MA, USA). Measurements were taken every 60 min and mean daily SWC (Vol. %) and soil temperature (°C) were calculated by averaging all measurements from three sensors.

### Dendrometer Records

In autumn 2013 we installed temperature compensated electronic dendrometers (Ecomatik, Munich, Germany) with resolutions of <3 μm. Band dendrometers (type DC2) and diameter dendrometers (type DD-S) were mounted on healthy mature trees at 1.3 m stem height (*n* = 6) and on saplings at 15 cm stem height (*n* = 7), respectively. The temperature coefficient of the sensor amounted to <0.1 μm/K. The measuring cable of band dendrometers consisted of Invar-steel, which shows a temperature coefficient of linear expansion <1 μm/mK. To reduce the influence of hygroscopic shrinking and swelling effects on dendrometer records (DMR), dead outermost layers of the bark (periderm) were slightly removed. Mean tree age of selected mature trees and saplings was estimated to be >100 year and <20 year, respectively (cf. Schuster and Oberhuber, 2013b). Data loggers were programmed to record measurements taken every 30 min and daily increments of stem radius were calculated by averaging all daily measurements (48 values/day). The Gompertz-function, which is an asymmetric sigmoid curve, i.e., it accelerates more quickly than it decelerates, was used for describing the long-term development of radial increment over the whole growing season (e.g., Rossi et al., 2003). To this end, the non-linear regression procedure included in the Origin software package (OriginLab Corporation, Northampton, MA, USA) was applied.

### Tree Water Deficit and MDS

**FIGURE 1:**
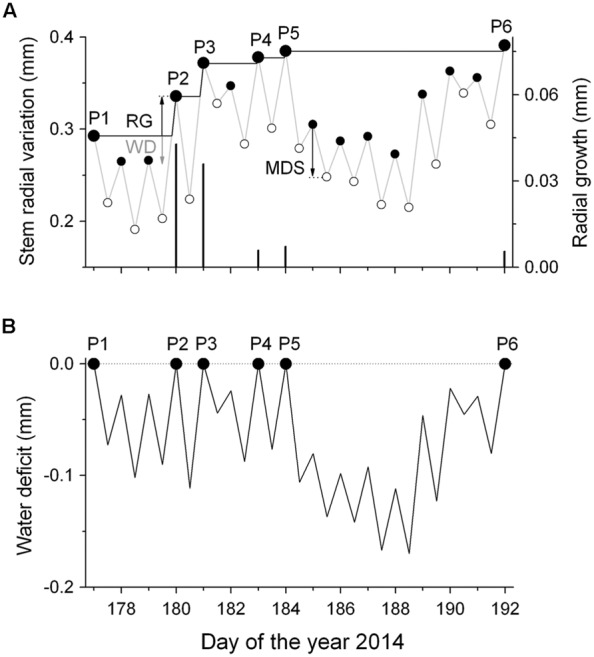
**Scheme of detrending dendrometer records (DMR) for growth. (A)** Time series of daily maximum and minimum DMR (gray line) and fitted ‘growth line’ (black line) (RG, radial growth; MDS, maximum daily shrinkage; WD, water deficit). The symbols P1–P6 indicate successively increasing daily maximum DMR, whereby the difference among successive values is regarded as RG (black bars). MDS is the diurnal amplitude between the morning maximum and the daily minimum (filled and open circles, respectively). **(B)** The extracted daily stem water deficits (ΔW) as the difference between the ‘growth line’ and the daily maximum and minimum DMR (small closed and open circles, respectively). Missing stem radius recovery indicates a continuous ΔW.

To distinguish between growth-related and water-status-related stem radius changes, DMR were de-trended for growth according to Ehrenberger et al. (2012). In particular, a ‘growth line’ was constructed by drawing a horizontal line from the daily maximum value to the next equal stem radius value ignoring periods of incomplete stem radius recovery due to drought induced stem shrinkage (**Figure 1A**). Then, the growth line followed the slope of the original DMR representing daily RG. This procedure was repeated until the end of the measurement period in early October. By applying this method it is assumed that RG, which requires an increase in cell volume and depends on high cell turgor pressure, is restricted to short periods when the stem is rehydrated rather than occurring constantly throughout the growing season (cf. Zweifel et al., 2005). In the following, tree water deficit (ΔW) was determined as the difference in stem size under low water availability conditions relative to the stem size under fully hydrated conditions (ΔW = 0). Hence, increasingly negative values of ΔW due to dehydration of storage pools indicate increasing drought stress (**Figure 1B**). Additionally, the magnitude of MDS (μm) defined as the difference between the daily maximum, and minimum stem radius (diurnal amplitude), was determined (**Figure 1A**). Although the maximum diameter was usually observed in the early morning, the minimum diameter was normally reached during the late afternoon, reflecting the daily cycle of water uptake and loss. Bark width (excluding periderm) was determined during a cool-moist period in the fall by sampling increment cores and stem disks from mature trees and saplings, respectively.

### Wavelet Analysis of DMR

Wavelet transform is used to decompose a time series over a time-scale space, thus providing visualization of a power distribution along time and frequency. It is a powerful tool to analyze non-stationary signals and it permits the detection of main periodicities in a time series and the evolution of their respective amplitude, frequency, and phase (Percival and Walden, 2000). We used the Morlet wavelet which is a sine wave modulated by a classical Gaussian function, because it establishes a clear distinction between random fluctuations and periodic regions (Torrence and Compo, 1998). Following Grinsted et al. (2004) the dimensionless frequency (ω_0_) was set to 6. The generated wavelet spectrum is a time-scale plot, where the x- and y-axis represent the position along time and periodicity scale, respectively, and the color contour at each x/y point represents the magnitude of the wavelet coefficient at that point. A dark red color is assigned to the highest value of the wavelet power spectrum, whereas a dark blue color is assigned to the lowest value. As continuous wavelet analyses are applied to the time series of finite length, edge effects may appear on the wavelet spectrum, leading to the definition of a cone of influence (Torrence and Compo, 1998), which is shown in a lighter shade in the wavelet spectrum. Statistical significance levels were estimated against a red noise model with lag-1 autocorrelations estimated from the observed time series (Grinsted et al., 2004). The wavelet analysis was performed on half-hourly DMR of saplings (*n* = 7) and mature trees (*n* = 5) from April through September after removing the long-term sigmoidal growth trend from the original series. Wavelet analyses were performed using the Matlab (Mathworks, Natick, MA, USA) wavelet toolbox of Torrence and Compo (1998), provided by Aslak Grinsted (http://www.mathworks.com/matlabcentral/fileexchange/47985-cross-wavelet-and-wavelet-coherence).

### Climate Influence on Tree Water Status and Growth

Pearson correlation statistics (*r*) were calculated to explore the relationship between daily time series of environmental variables for the period of April–October (precipitation, RH, vapour pressure deficit (VPD), air and soil temperature, SWC and time series of ΔW, MDS, and RG extracted from DMR. Kolmogorov–Smirnov tests were applied to check for the normal distribution of selected variables. The Spearman rank-correlation coefficient (ρ) was determined for non-normally distributed variables. Temporal changes in these relationships were evaluated by moving correlation analysis (MCA), which is based on progressively shifting the period of a fixed number of days across time to compute the correlation coefficients. To provide robust measures of association between environmental variables and ΔW, MDS, and RG, the length of the calibration window was set to 30 days. Correlation coefficients were arbitrarily plotted against the last day of the period. The software packages used for the analyses were Statistica, version 8.0 (StatSoft, Inc., Tulsa, OK, USA) and Matlab R2010b.

## Results

### Environmental Variables during the 2014 Growing Season

**FIGURE 2:**
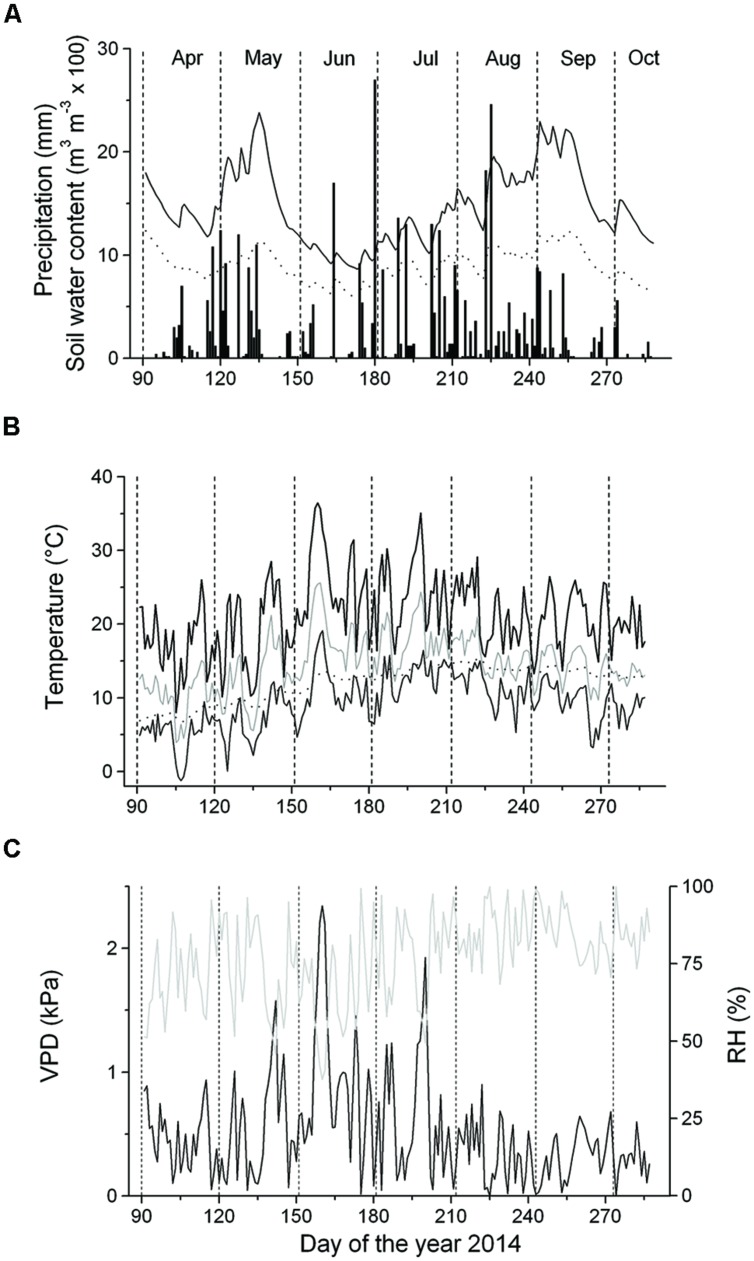
**Climate variables and soil water content (SWC) recorded during the growing season 2014. (A)** Daily precipitation sum (bars) and SWC at the 5–10 cm and 10–15 cm soil depth layers (solid and dashed line, respectively); **(B)** Daily mean (gray line), minimum (thin solid line) and maximum air temperature (thick solid line) and mean daily soil temperature (dotted line); **(C)** Vapor pressure deficit (VPD, solid line) and relative air humidity (RH, gray line).

At the experimental site, monthly precipitation ranged from 39 mm (September) to 94 mm (August) (**Figure 2A**). Starting with frequent rainfall events in mid-April, SWC reached c. 0.25 m^3^ m^-3^ until mid-May, when the occurrence of sporadic low rainfall events caused a decrease of SWC to c. 0.10 m^3^ m^-3^ in the 5–10 cm soil depth layer for several weeks. Frequent rainfall events in late-June through early September increased SWC to c. 0.25 m^3^ m^-3^ in the 5–10 cm soil depth layer (**Figure 2A**). Monthly mean temperature ranged from 10.4°C (April) to 17.7°C (July), with an overall mean value of 14.7°C during April–September. Maximum air temperature reached 36.4°C in early-June. Daily mean soil temperature in the 5–10 cm soil depth layer generally followed the trend of air temperature with minor amplitudes developed in the former (**Figure 2B**). Mean VPD amounted to 0.53 kPa from April to September and the VPD maxima reached 2.3 kPa in early June (**Figure 2C**). VPD was not significantly different above and below the canopy (data not shown), which is most likely caused by a fairly open canopy of the selected study plot (canopy coverage is 70%).

### Stem Radius Changes and Dendrometer-Derived Tree Water Deficit

**FIGURE 3:**
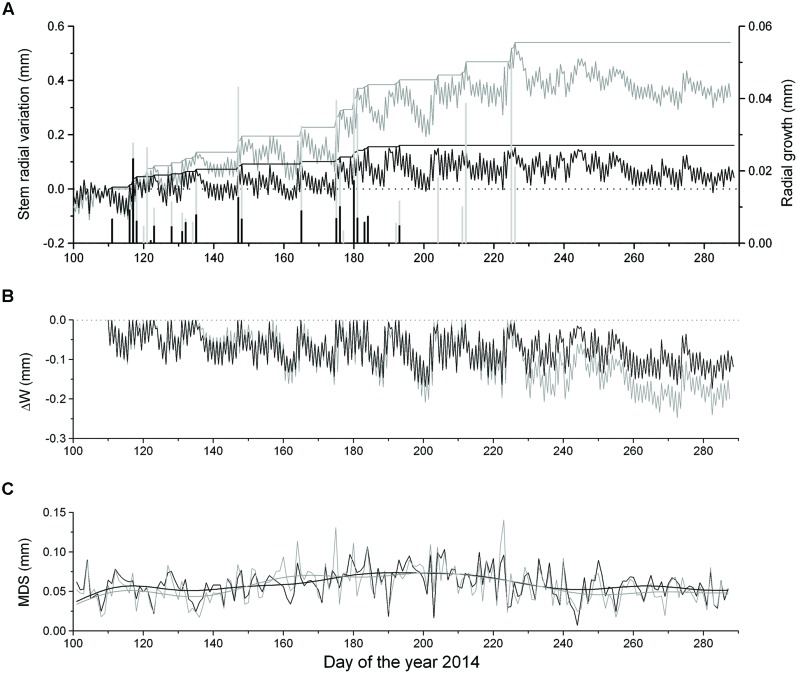
**(A)** Time series of maximum and minimum daily DMR, growth line, and extracted RG (bars) of saplings (solid lines and bars) and mature trees (gray lines and bars), **(B)** ΔW of saplings (solid line) and mature trees (gray line) and **(C)** maximum daily shrinkage (MDS) of saplings (solid line) and mature trees (gray line). Data were smoothed based on a fast Fourier transform low-pass filter, whereby the number of points was set to 20.

Dendrometer traces of saplings and mature *P. abies* showing daily maximum and minimum values are depicted in **Figure 3A**. Cumulated daily RG amounted to 0.54 mm ± 0.14 and 0.16 mm ± 0.03 in mature trees and saplings, respectively, indicating radial increments more than three times in the former in 2014. Extracted daily RG was consistently lower in saplings than mature trees. RG occurred mostly concurrently in saplings and mature trees during periods of frequent rainfall (cf. **Figures 2A** and **3A**). Modeling DMR using Gompertz functions revealed that intra-annual RG peaked in mid-May (doy 137) and early June (doy 153) in saplings and mature trees, respectively (data not shown). RG stopped in mid-July (doy 193) in saplings and mid-August (doy 226) in mature trees (**Figures 3A,B**).

Extracted ΔW showed synchronous fluctuations among saplings and mature trees and reached –0.15 mm in saplings and –0.21 mm in mature trees, when incomplete rehydration was detected during the growing season (**Figure 3**). After the end of RG ΔW did not recover by mid-October indicating a permanent ΔW in both groups. Maximum ΔW values were determined in late September and corresponded to 9.4 and 6.9% of living tissues of the bark of saplings and mature trees, respectively. Bark width was twice as great in mature trees compared to saplings (**Table 1**). Mean MDS values amounted to 57 ± 22 μm and 59 ± 19 μm in mature trees and saplings, respectively (**Figure 3C**) and were not significantly different among groups. A slightly increasing trend in MDS was detected in both series during summer (June–August), when higher temperatures prevailed.

**FIGURE 4:**
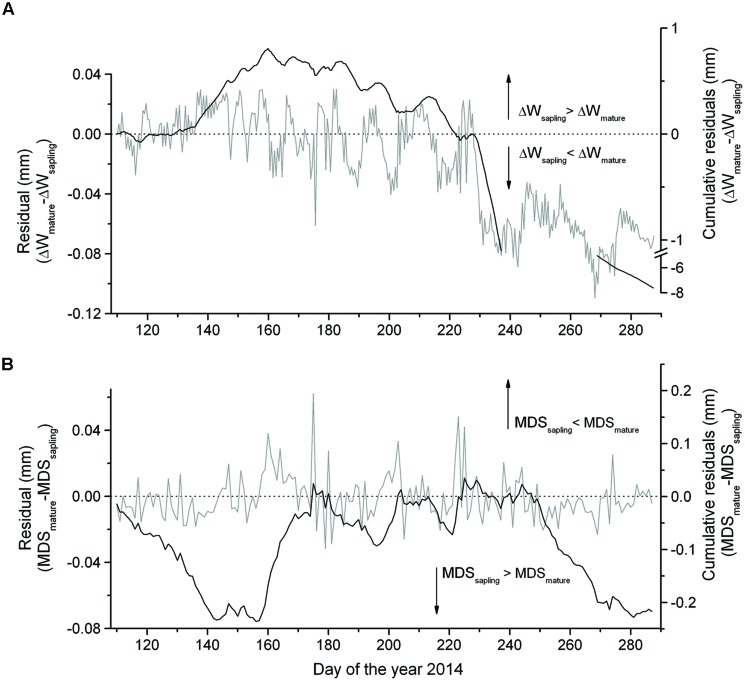
**Difference in the **(A)** ΔW and **(B)** MDS between mature trees and saplings and cumulative residuals from mid-April through early October 2014**.

**FIGURE 5:**
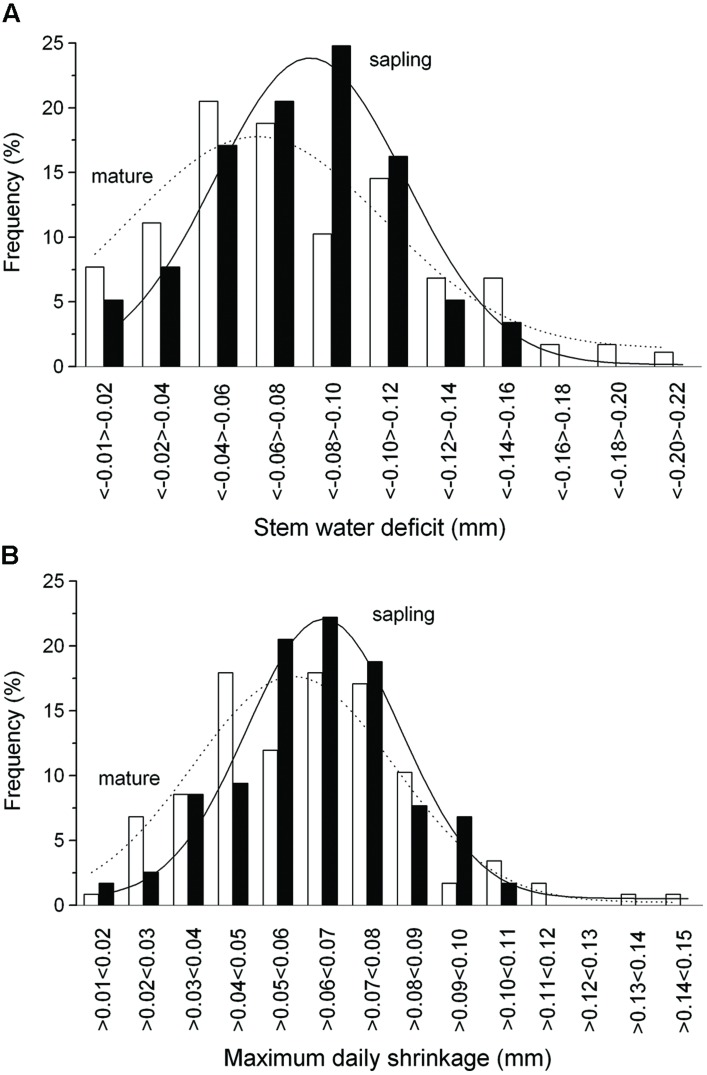
**Frequency distribution of the ΔW **(A)** and MDS **(B)** in mature trees (open bars) and saplings (filled bars)**. Solid and dashed lines indicate Gaussian-fitting for saplings and mature trees, respectively.

The summation of the residuals of ΔW_mature_ – ΔW_sapling_ and MDS_mature_ – MDS_sapling_ revealed that starting with a period of low precipitation in mid-May and decreasing SWC (**Figure 2A**) saplings developed higher ΔW and MDS than mature trees (**Figure 4**). Secondly, the former relationship slowly decreased with increasing precipitation and SWC at end of June and abruptly reversed after mid-August (doy 226), when permanently higher ΔW was developed in mature trees than saplings. Cumulative residuals of MDS, however, leveled off more quickly with the occurrence of higher precipitation events. Higher ΔW and MDS in saplings compared to mature trees is also indicated by higher frequencies of more negative ΔW and large MDS values in saplings than mature trees (**Figures 5A,B**).

**FIGURE 6:**
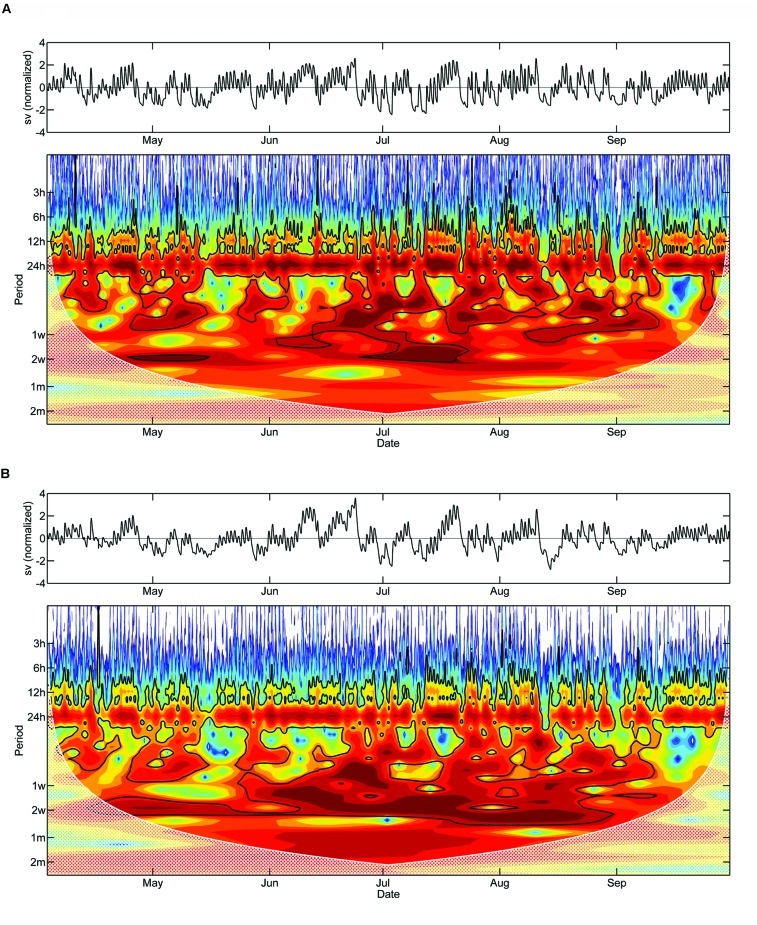
**Detrended stem variations and Morlet wavelet spectra of half-hourly DMR of saplings **(A)** and mature adult trees (B).** The black line outlines designate the 5% significance level. The ‘cone of influence,’ where edge effects become important and the results should be ignored, are shown in a lighter shade. Red and blue colors indicate high and low wavelet power spectrum values, respectively (sv, stem variation).

Morlet wavelet analysis on the detrended DMR of saplings and mature trees revealed a statistically significant daily cycle (*P* < 0.05) during a large part of the study period (**Figures 6A,B**). The diurnal cycle is, however, notably less pronounced in the latter. Significant periodicities of several days up to 2 weeks also occurred in both wavelet spectra. While these periodicities are localized in time for the saplings, periodicities of approximately 2 weeks occurred almost continuously over the study period in mature trees.

### Influence of Environmental Variables on Tree Water Deficit and Growth

**FIGURE 7:**
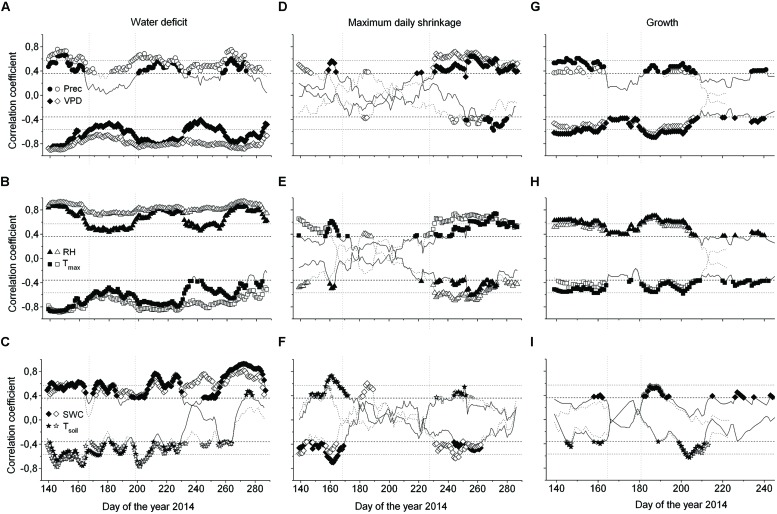
**Moving correlations (window 30 days) between the water deficit (ΔW) **(A–C)**, MDS **(D–F)** and growth **(G–I)**, and environmental variables of saplings (open symbols) and mature *Picea abies* (filled symbols).** Spearman rank-correlations (ρ) were calculated for relationships between (i) precipitation and ΔW, MDS, and growth, and (ii) all environmental variables and growth. For all other relationships the Pearson product-moment coefficients (*r*) are shown (Prec, daily precipitation sum; VPD, vapor pressure deficit; RH, relative air humidity; T_max_, daily maximum air temperature; SWC, soil water content; T_soil_, mean soil temperature). A lag of 1 day was considered in the relationship between ΔW, MDS, and growth, and SWC and T_soil_. Horizontal dotted and dashed lines indicate *P* = 0.001 and *P* = 0.05 significance levels, respectively.

Considering the whole study period, i.e., from late April through early October, all environmental variables were highly significantly correlated with ΔW of saplings (**Table 2**), whereby air and soil temperature and VPD (inverse relationships) and precipitation (direct relationship) showed the highest correlation coefficients. MCA applying a window of 30 days indicated largely temporal stability of these relationships throughout the study period (**Figures 7A–C**). In mature trees, however, only precipitation and air and soil temperature showed significant relationships with ΔW over the whole study period, whereas MCA showed almost continuous significant relationships for VPD, RH and SWC (**Figures 7A–C**).

**Table 2:** Correlation coefficients [Pearson product-moment (*r*) and Spearman rank-correlation coefficient (ρ)] between environmental variables (Prec, precipation; SWC, soil water content; VPD, vapor pressure deficit; RH, relative humidity; Tsoil, mean soil temperature; Tair_min_, minimum air temperature; Tair_mean_, mean air temperature; Tair_max_, maximum air temperature) and water deficit (ΔW), maximum daily shrinkage (MDS) and growth of saplings and mature *Picea abies*.

	Sapling			Mature		
	ΔW	MDS	Growth^1^	ΔW	MDS	Growth^1^
Prec^1^	0.501***	–0.092	0.373***	0.272***	–0.091	0.345***
SWC	0.350***	–0.285***	0.189	0.074	–0.370***	0.216*
VPD	–0.450***	0.230**	–0.522***	–0.077	0.278***	–0.511***
RH	0.427***	–0.208**	0.557***	0.026	–0.177*	0.503***
Tsoil	–0.462***	0.194***	–0.140	–0.514***	0.255***	–0.134
Tair_min_	–0.250***	0.145	–0.113	–0.141	0.324***	–0.175
Tair_mean_	–0.508***	0.344***	–0.349***	–0.219**	0.435***	–0.394***
Tair_max_	–0.583***	0.426***	–0.379***	–0.261***	0.408***	–0.437***

A one-day lag in ΔW, MDS, and growth was considered in correlations with SWC and Tsoil (ΔW and MDS: n = 178 for both, saplings and mature trees; lag1: n = 177; growth: n = 84 and 117 for saplings and mature trees, respectively). *P ≤ 0.05; **P ≤ 0.01; ***P ≤ 0.001. ^1^Spearman rank-correlation coefficient (ρ).

Taking the whole study period into account, MDS showed the closest correlations with air temperature and SWC (direct and inverse relationship, respectively) in mature trees and saplings (**Table 2**). MCA, however, revealed decreased sensitivity of MDS to all environmental variables during periods of high rainfall occurring from July through August (**Figures 7D–F**). Although stem radial increments extracted from DMR were highly significantly related to VPD, RH, air temperature, and precipitation in saplings and mature trees (**Table 2**), MCA indicated temporal instability of these relationships (**Figures 7G–I**). Overall, based on MCA saplings and mature trees showed quite similar response of ΔW, MDS, and growth to environmental variables, and predominantly closer correlations were found using a 30 day window compared to taking the whole study period, i.e., late-April to early October for ΔW and MDS, or the growing season, i.e., late-April through July, into account (**Figure 7**; **Table 2**).

## Discussion

Total increment in 2014 differed more than threefold among saplings and mature trees, which is consistent with a previous study that showed that RG of young *P. abies* within the study area is strikingly suppressed compared to mature adult trees (Schuster and Oberhuber, 2013b). Furthermore, DMR revealed that saplings reached maximum growth earlier and also RG stopped earlier compared to mature trees. Because it was frequently found that water stress causes earlier culmination and ending of RG in *P. abies* (Pichler and Oberhuber, 2007; Levanič et al., 2009; Swidrak et al., 2014) and other conifers (e.g., Gruber et al., 2010), we relate the differences in annual radial increments among saplings and mature trees primarily to the development of adverse stem water status in small trees. Our reasoning is supported by findings that water deficits affect growth earlier and more intensively than photosynthesis, i.e., sink limitation rather than source limitation of growth prevails under water deficits (for a review see Muller et al., 2011). Anderegg and Anderegg (2013) also found no significant changes in carbohydrate concentrations in saplings of two conifer species during severe experimental drought.

### Stem Water Deficit in Saplings and Mature Trees

Results of this study are in contrast with our first hypothesis that a less distinct ΔW is developed in saplings compared to mature trees. Cumulative residuals and frequency distributions of ΔW and MDS indicate that higher amounts of water reserves were recruited in saplings than in mature trees to sustain leaf transpiration. This result implies that during a diurnal period, saplings more strongly exploit their internal stem water reserves than mature trees. This effect is confirmed by wavelet analysis, which revealed that the daily cycle of stem shrinking and swelling is more distinct in saplings than mature trees, i.e., greater daily amplitudes are developed in the former. The daily amplitude is related to environmental variables that influence the amount and rate of daily transpiration, as reported by King et al. (2013). Furthermore, the dominance of longer-term cycles (1 week up to 2 weeks) in mature adult trees suggests that more balanced water relations exist in larger trees. Cumulative residuals (ΔW_mature_ – ΔW_saplings_) reached maximum values in June–July when SWC was at a minimum and the highest temperatures were recorded. High evaporative demand and long day length during a period of low SWC less strongly influenced the ΔW of mature trees, i.e., an insufficient replenishment of water in the expandable tissues led to higher ΔW in saplings compared to mature trees. Our findings revealed that residuals of MDS showed an earlier response than residual ΔW, which is consistent with reports of several authors that an increase in MDS is the first detectable morpho-physiological signal of changes in water status of the whole plant (e.g., Conejero et al., 2007; Giovannelli et al., 2007).

We suggest that a higher ΔW found in saplings compared to mature trees throughout the study period is most likely related to less efficient or competitive water uptake, i.e., mature trees have developed a more extensive root network (Mueller et al., 2005; Voelker, 2011) close to the soil surface, where SWC throughout the growing season was found to be constantly higher than in deeper soil layers. Schmid and Kazda (2002) reported that fine roots of *P. abies* are distributed primarily in upper soil layers. Furthermore, Phillips et al. (2003) found significantly greater ratios of sapwood volume to leaf area in large compared to small trees indicating that water storage capacity relative to water use increases with tree size. This corresponds to results of our study that ΔW was higher in saplings than mature trees.

Conversely, although *P. abies* is known to maintain a stable leaf water status over a wide range of evaporative demand or SWC through stomatal control, i.e., isohydric behavior (Buckley, 2005; Zweifel et al., 2009; Leo et al., 2014), saplings might have contrasting hydraulic behavior from mature trees. In saplings high transpiration rates presumably occur under moderate drought to maintain carbon assimilation at higher rates as SWC decreases (‘anisohydric’ behavior). This assumption is consistent with findings of Cinnirella et al. (2002), who found isohydric behavior in mature *Pinus laricio*, which was not observed in potted seedlings subjected to drought. Furthermore, it has to be considered that the same individual can switch from isohydric behavior to anisohydric behavior in response to changing SWC (Domec and Johnson, 2012). Differences in size of the shrinking tissue, in the conductance of water between the bark and the xylem vessels and in RG can also affect dynamics of ΔW (Waring et al., 1979; Naor and Cohen, 2003) and MDS (Intrigliolo and Castel, 2005; Dawes et al., 2014). Specifically, significantly lower phloem thickness in saplings than mature trees may restrict stem shrinkage in the former, while significantly higher RG found in mature trees compared to saplings may more strongly mask stem shrinkage in mature trees.

### Environmental Control of Tree Water Status and RG

Correlation analyses revealed that ΔW of saplings is more strongly affected by environmental conditions than ΔW of mature trees throughout the study period. This finding is in line with the development of higher ΔW in saplings compared to mature trees. Conversely, MDS of both saplings and mature trees showed quite similar responses to environmental variables, and low stability between environmental factors and MDS was detected. We explain this finding by the relocation of water from storage locations (sapwood, cell walls, and inactive vessels) to the elastic tissues of the bark, which might contribute to temporary decoupling of environmental variables especially from MDS (cf. Zweifel et al., 2000). Hence, results of this study are only partly consistent with our hypothesis that both stem water indicators are most closely related to climate variables that influence transpiration. Instability of the relationship between stem water indicators and environmental variables, which is in contrast to our expectation, can be explained by the alternation of unfavorable and favorable environmental conditions throughout the study period and high sensitivity of especially MDS to water availability. Similarly, Vieira et al. (2013) found varying climatic response of dendrometer derived water status parameters and radial increments in *Pinus pinaster* in a drought-prone environment. Therefore, MCA should be applied when evaluating growth and/or stem water status parameters in response to environmental variables that show high variability throughout the growing season. Coupling of ΔW and MDS to atmospheric conditions indicates that transpiration strongly draws upon water reserves from the living bark, especially of young trees. This observation is in line with several other studies (e.g., Zweifel et al., 2005; Turcotte et al., 2011; Ehrenberger et al., 2012). On the other hand, mature trees showed no significant correlation between ΔW and SWC, VPD, and RH indicating that large trees have a strong ability to regulate drought stress, because of a more extensive root system and/or greater water storage capacity. The lag observed between soil variables (SWC, soil temperature) and ΔW and MDS indicate a time lag between water loss by transpiration and refilling of water reserves in the stem (Meinzer et al., 2004). Higher sensitivity of ΔW to environmental variables compared to MDS is most likely related to the method in, which ΔW is calculated, i.e., periods of missing stem rehydration are represented in ΔW but not in MDS.

Although RG was found to be closely related to atmospheric conditions, soil parameters (SWC, soil temperature) had a marginal effect on growth. This effect is consistent with findings in previous studies at low (Oberhuber et al., 2014) and high altitude (Gruber et al., 2009), in which a close coupling of RG of mature deciduous and evergreen coniferous species to atmospheric conditions was detected. These findings imply that transpiration draws upon internal storage tissues in the stem rather than soil water (Čermak et al., 2007; Betsch et al., 2011) and in this way prevents low stem water potentials that might be caused by peaks of transpiration. As a result, high turgor in cambial cells and their differentiating derivatives favor cell division and cell enlargement. In a dendrometer study Köcher et al. (2012) also reported that atmospheric moisture status rather than other hydrological variables, especially precipitation and soil moisture, influenced cambial growth of five temperate broad-leafed tree species.

Notably similar RG responses of saplings and mature trees to environmental variables were detected in this study, which is in contrast to long-term analysis of climate-growth relationships in dendroclimatological studies revealing higher climate sensitivity of older and larger *P. abies* trees (Schuster and Oberhuber, 2013b). These contrasting results indicate the influence of time scale (days vs. years) on RG response to environmental conditions. Most likely, several seasonal growth phases are integrated in annual increments (i.e., tree ring width), which might affect the climate-growth relationship, whereas high-resolution DMR allows for studying environmental factors on RG directly. Furthermore, different autocorrelation signals in inter-annual (tree rings) vs. intra-annual (DMR) time series might exist.

Results of this study revealed a more strained stem water status in saplings as opposed to mature *P. abies* trees in response to limited water availability and evaporative demand during the growing season. Size-related differences in root morphology and physiology and hydraulic capacitance (cf. Scholz et al., 2011) under drought are needed to prove the suggested processes. We conclude that productivity, structure and distribution of drought-prone Norway spruce forests will be increasingly affected if in the dry inner-Alps the projected decrease in water availability develops in future decades (Christensen et al., 2007).

## Author Contributions

WO assembled data, made conception of the paper, coordinated the research project and drafted the article; all authors analyzed and interpreted the data; AH and WK discussed and commented on the manuscript.

## Conflict of Interest Statement

The authors declare that the research was conducted in the absence of any commercial or financial relationships that could be construed as a potential conflict of interest.
